# Syphilis self-testing to expand test uptake among men who have sex with men: a theoretically informed mixed methods study in Zimbabwe

**DOI:** 10.1136/sextrans-2020-054911

**Published:** 2021-04-29

**Authors:** Clarisse Sri-Pathmanathan, Definate Nhamo, Takudzwa Mamvuto, Gwendoline Chapwanya, Fern Terris-Prestholt, Imelda Mahaka, Michael Marks, Joseph D Tucker

**Affiliations:** 1 Faculty of Infectious and Tropical Diseases, London School of Hygiene & Tropical Medicine, London, UK; 2 Pangaea Zimbabwe AIDS Trust, Harare, Zimbabwe; 3 Faculty of Public Health and Policy, London School of Hygiene & Tropical Medicine, London, UK; 4 Institute of Global Health and Infectious Diseases, University of North Carolina at Chapel Hill, Chapel Hill, North Carolina, USA

**Keywords:** syphilis, diagnostic techniques and procedures, homosexuality, male, developing countries, sexual health

## Abstract

**Objectives:**

Self-testing for STIs such as HIV and syphilis may empower sexual minorities and expand uptake of STI testing. While much is known about HIV self-testing (HIVST), less is known about syphilis self-testing, particularly in low-income settings. The objective of this study is to determine context-specific facilitators and barriers for self-testing and to assess the usability of syphilis self-testing in Zimbabwe among men who have sex with men (MSM).

**Methods:**

This mixed methods study was conducted in Harare as part of a larger syphilis self-testing trial. The study included in-depth interviews (phase I) followed by usability testing and a second interview (phase II). In-depth interviews were conducted with MSM and key informants prior to syphilis self-testing. The same MSM then used the syphilis self-test, quantitatively assessed its usability and participated in a second in-depth interview. Phase I data were analysed using a thematic approach, guided by an adapted social ecological model conceptual framework. Phase II interviews were analysed using rapid assessment procedure methodology, and usability was assessed using a pre-established index, adapted from existing HIVST scales.

**Results:**

Twenty MSM and 10 key informants were recruited for phase I in-depth interviews, and 16 of these MSM participated in phase II by completing a syphilis self-test kit. Facilitating factors for self-testing included the potential for increased privacy, convenience, autonomy, and avoidance of social and healthcare provider stigma. Barriers included the fear to test and uncertainty about linkage to care and treatment. Data from the Usability Index suggested high usability (89.6% on a 0–100 scale) among the men who received the self-test.

**Conclusions:**

MSM in Zimbabwe were willing to use syphilis self-test kits and many of the barriers and facilitators were similar to those observed for HIVST. Syphilis self-testing may increase syphilis test uptake among sexual minorities in Zimbabwe and other low-income and middle-income countries.

## Introduction

In 2016, the WHO estimated 19.9 million cases of syphilis worldwide, with the highest prevalence in the WHO African region.^1^ In the same year, the Global Health Sector Strategy on Sexually Transmitted Infections set an impact goal to reduce syphilis infections by 90% globally between 2018 and 2030. As syphilis is often asymptomatic, testing is essential to effectively interrupt transmission, and innovative strategies are needed to expand syphilis test uptake.^2^ Syphilis is more common among men who have sex with men (MSM), with the WHO reporting a median seroprevalence of 6.0% in this group, estimated from 2016 to 2017 Global AIDS Monitoring data.^3^ A 2020 biobehavioural survey in Zimbabwe found that 5.1% of Harare MSM had positive treponemal and non-treponemal tests.^4^ In addition, syphilis and HIV share common sexual risk behaviours, and syphilis facilitates HIV transmission, making syphilis coinfection particularly prevalent in HIV-infected MSM.^2 5^ The biobehavioural survey reported a 12.7% prevalence of active syphilis coinfection among HIV-infected MSM in Harare.^4^ As a result, the WHO strongly recommends routine syphilis screening among MSM.^6^

MSM are often prevented from accessing sexual health services because of lack of funding, lack of testing, legal and cultural barriers, and stigmatisation, particularly in low-income and middle-income countries (LMICs).^7^ In Zimbabwe, same-sex relations are criminalised, with a penalty of 1-year imprisonment and a fine. Additionally, being openly gay is culturally taboo, and this stigma encourages many MSM to not disclose their sexual orientation and to engage in heterosexual marriages. Research shows that stigma associated with same-sex relationships also extends to healthcare facilities and professionals serving MSM.^8^ There is a considerable gap in evidence to guide MSM health programmes in many LMICs.^9^ As a result, despite WHO recommendations, MSM are frequently excluded from syphilis testing services in Zimbabwe.^2^

One way to expand MSM syphilis test uptake is self-testing. Syphilis self-testing is an approach whereby a person performs a rapid test and interprets the result in private. Self-testing may overcome some of the barriers associated with facility-based testing, promoting early diagnosis and interrupting disease progression.^10^ This method has been explored in China and The Netherlands, where syphilis self-testing was feasible.^11 12^

HIV self-testing (HIVST) is recommended by the WHO to expand test uptake among stigmatised key populations.^6^ A qualitative evidence synthesis found that HIVST empowered people and decreased test-associated stigma.^13^ Many countries, including Zimbabwe, have policies to support HIVST as an entry point into sexual health services.^14^ However, there is less evidence supporting syphilis self-testing, despite the known importance of qualitative research in implementing novel diagnostic technologies.^13^ Syphilis self-testing pilots have shown that it may increase testing frequency by empowering MSM and reducing the impact of structural barriers, but there are no data from sub-Saharan Africa.^11 15^ Additionally, in the context of the COVID-19 pandemic, self-testing has become an increasingly important pathway to safely sustain testing when testing facilities are closed or only partially open.

This study aims to understand how syphilis self-testing can create opportunities to test for MSM in Zimbabwe. The purpose of this study was to determine facilitators and barriers for syphilis self-testing and to assess the usability of syphilis self-testing as reported by Zimbabwean MSM.

## Methods

A two-phased mixed methods study was conducted among MSM in Harare due to the strong network of MSM community-based organisations in the city. The first phase was prior to testing, and the second phase was after syphilis self-testing. Formative data from both phases informed a trial protocol comparing syphilis self-testing to facility-based testing in MSM in Zimbabwe.^16^

In phase I, in-depth interviews were conducted among MSM and key informants, by trained researchers from the Pangaea Zimbabwe Aids Trust (PZAT), between March and April 2020. We recruited participants using snowball sampling until we reached saturation of themes. Participants had to be 16 years or older, living in Harare, ever had anal or oral sex with another man, born biologically male and able to provide informed consent.^17^ All MSM recruited were referral facilitators, offering community support to other harder-to-reach MSM and were not remunerated. Key informants were healthcare professionals and were purposively sampled to include providers who had experience with HIV and/or syphilis testing.

Interviews lasted 30 min and were audio-recorded. The MSM interview guide was developed to collect sociodemographic data, explore prior syphilis-testing and HIV-testing experiences, facilitating and deterring factors, and self-testing intervention preferences (online supplemental file 1). The key informant interview guide included healthcare provider experiences and challenges with sexual health services, and populations served.

10.1136/sextrans-2020-054911.supp1 Supplementary data

**Figure 1:**
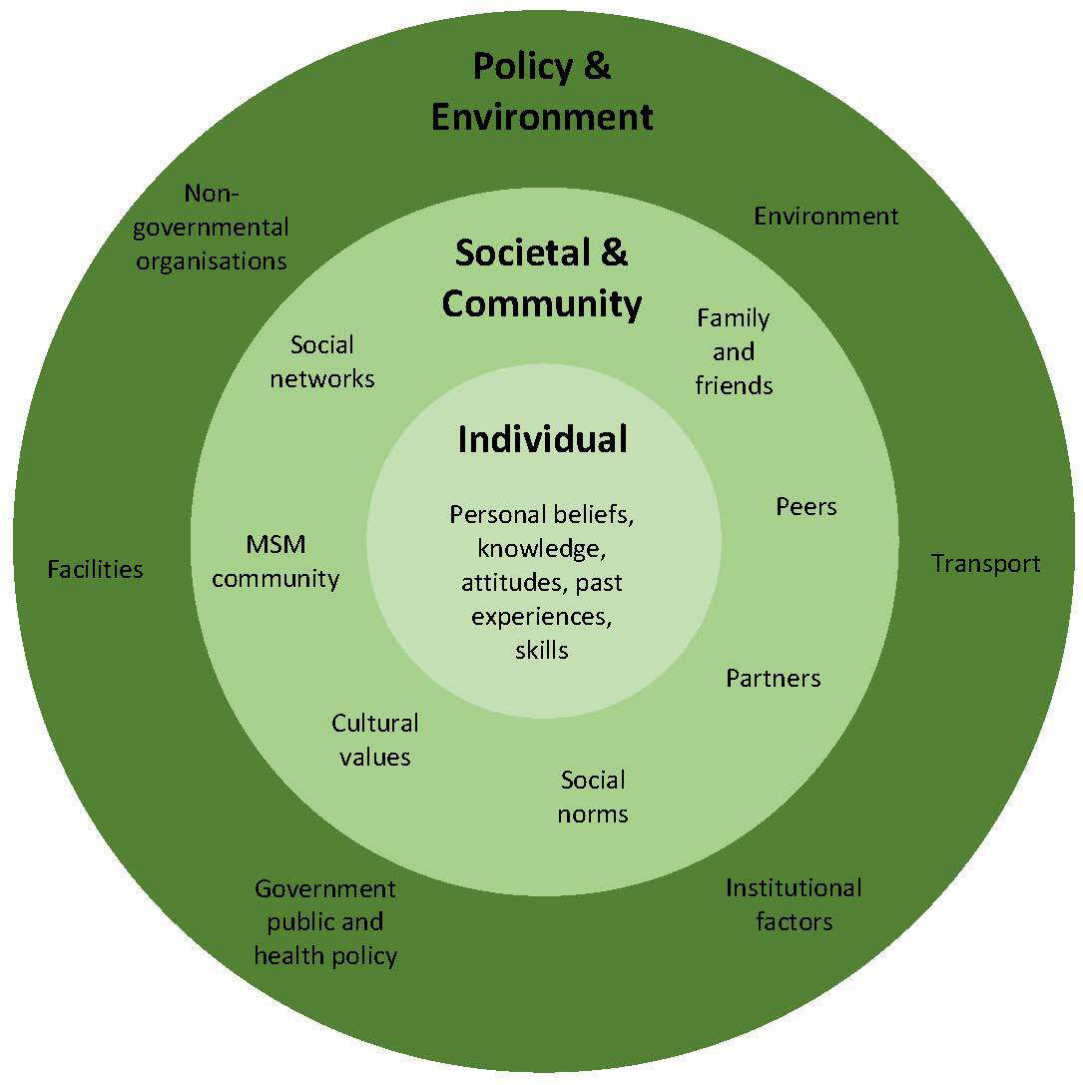
An adapted social–ecological framework of factors influencing test uptake and acceptability of a syphilis self-testing intervention among MSM.^19^ MSM men who have sex with men.

Interviews were translated and transcribed by PZAT researchers. Transcripts were entered into Dedoose 8.3.17. The Framework Method was used to guide our analysis.^18^ Two codebooks were developed based on an adapted social–ecological model to systematically analyse the data. Ultimately, our conceptual framework included an individual level, a community level and a policy and environment level (figure 1).^19^ The framework was used to organise deductive and inductive themes emerging from the data, and to create separate analytical memos for MSM and key informant data. The preliminary findings described in these analytical memos were used to refine the pilot trial protocol (MRCZ/A/2533).

In phase II, the syphilis self-test distributed to MSM consisted of a Standard Q Syphilis Ab treponemal blood-based rapid test, adapted for individual use and interpretation. SD Biosensor reports 100% sensitivity and 97.5% specificity of the antibody test, with a result appearing within 5–20 min. This is a lateral flow immunochromatographic assay similar to blood-based HIVST kits. Individual lancets and buffer samples were packaged into sealed pouches, with an individual test device and infographic, detailing step-by-step use, disposal and procedure for confirmatory testing. An instructional video was created and disseminated to facilitate independent use. Tests were distributed by researchers from PZAT to the same MSM who had completed phase I.

In phase II (August 2020), PZAT researchers interviewed a sample of 16 MSM who successfully completed a self-test. Interviews were conducted under COVID-19 social distancing measures. An exit interview guide was developed to qualitatively assess specific facilitators and barriers for syphilis self-testing. Participants also completed a 15-item Usability Index (UI) to assess the usability of the test, adapted from a study of HIVST usability in South Africa. The index includes dichotomous questions about specific items related to the process of self-testing. Like in the HIVST study, we tracked all successful steps in completing the self-test, in order to quantify a UI, expressed as a percentage.^20^ Qualitative data from the survey was analysed following the rapid assessment procedures (RAP), a methodology designed for rapid assessment of health-seeking behaviour.^21^ Data were organised into a RAP matrix by paraphrasing and synthesising participant responses. The template can be found in online supplemental file 3. This allowed us to systematically identify similarities, differences and trends in responses.^22^

10.1136/sextrans-2020-054911.supp3 Supplementary data

## Results

Twenty MSM and 10 key informants were recruited for phase I in-depth interviews. In phase II, 16 MSM were invited to conduct the self-test and were subsequently interviewed. Four were lost to follow-up due to relocation or communication difficulties. Upon testing, it emerged that insufficient quantities of buffer were provided in some kits. This was resolved through community-based distribution of additional buffer samples.

In phase I, 18 of 20 MSM had previously used HIVST (table 1). All participants had at least secondary-level education, and all but three self-identified as gay. We observed the following themes in qualitative data: prior STI and HIV testing experiences, both with self-testing and facility-based services; usability of the syphilis self-test and how it compares to HIVST; MSM-specific facilitators and barriers for self-testing.

**Table 1:** Demographic characteristics of in-depth interview participants and exit interviews following the syphilis self-test kit trial

	Men who have sex with men n (%)
Phase I: in-depth interview	
Age	
Mean age in years (range)	24 (20–33)
Education level	
Secondary	7 (35.0)
College	6 (30.0)
University	7 (35.0)
Employment status	
Student	7 (35.0)
Formal work	7 (35.0)
Informal work	3 (15.0)
Unemployed	2 (10.0)
Other	1 (5.0)
Sexual orientation	
Gay	17 (85.0)
Heterosexual	2 (10.0)
Bisexual	1 (5.0)
Self-reported disclosure of sexual identity	
Disclosed to family, friends or doctors	19 (95.0)
Not disclosed	1 (5.0)
History of HIV self-testing	
Yes	18 (90.0)
No	2 (10.0)
Phase II: syphilis self-testing exit interview	
History of syphilis facility-based testing	8 (50.0)
Conducted a syphilis self-test	16 (80.0)
Tested positive for syphilis	2 (12.5)
Confirmation of test result	
In person via community-based organisation	6 (37.5)
Through photo messaging via WhatsApp	9 (56.25)

### Prior HIV and STI testing experiences

In phase I, 18 MSM had experienced HIVST before using the oral HIV test. Ten participants stated they used HIVST every 3–6 months. In addition, 13 of the 18 HIVST-experienced MSM had then attended a facility and were empowered to seek facility-based services. Key informants confirmed that syphilis testing is usually reserved for pregnant women, and only three underwent training on how to work with MSM, suggesting MSM are largely neglected by STI services. Some providers recommended syphilis testing should be mandatory for key populations.

### Syphilis self-test usability and comparison with HIVST

Of the 16 participants in phase II, two (13%) tested positive for syphilis. One had a history of previous treatment and was offered a confirmatory non-treponemal test that was negative. The other had a new syphilis diagnosis and received a positive non-treponemal test, followed by a course of benzathine penicillin. Fifteen participants reported the clarity of explanations provided in the infographic and video was instrumental to successful test completion. Overall, MSM reported 89.6% usability for the syphilis self-test on a 0–100 scale. This is described in detail in table 2. The main challenge with the test kit, reported by 11 of the 16 participants, was the blood draw using the capillary pipette. Participants nonetheless felt this challenge was warranted for the test to function. One participant had difficulties extracting the buffer because insufficient quantities were provided. Four participants had to repeat the test, as they did not provide enough blood for the test to show a result.

**Table 2:** UI of the syphilis self-test based on a stepwise questionnaire administered in phase II

Usability checklist	Yes n	No n	UI (%)*
Did you find it easy to read/use the information sheet?	16	0	100
Did you find it easy to watch/use the instructional video?	16	0	100
Was it difficult for you to remove the kit components from the pack?	1	15	94
Did you verify that the silica gel pouch was yellow, to confirm their test was valid for use?	16	0	100
Did you remove the yellow shield from the lancet?	16	0	100
Did you have difficulty lancing (pricking) their finger using the blue lancet?	1	15	94
Did you have difficulty forming a blood droplet?	5	11	69
Were you able to pick up a blood drop up to the black line of the capillary pipette?	5	11	31
Were you able to open the green buffer bottle?	16	0	100
Were you able to use the pink pipette to pick up the buffer?	15	1	94
Did you drop three drops into the test device well?	15	1	94
Was a control line present on the test device?	12	4	75
Did you trust the self-test result?	15	1	94
Did you quit the process at any point?	0	16	100
Did you continue the process despite a missed or incorrect step?	0	16	100
Total UI (%)			89.6

*The UI was calculated based on the method used in the HIVST paper from which the index was extracted.^20^

HIVST, HIV self-testing; UI, Usability Index.

### Comparing syphilis self-testing to HIVST

Phase II participants felt that the syphilis and HIV self-test kits had many similarities, including the potential for privacy and convenience. The major challenge cited was that syphilis self-testing uses a blood sample, while most HIVST kits use oral samples. Two MSM reported a preference for HIVST compared with syphilis self-testing because of this issue. However, 15 (94%) participants felt that they trusted the syphilis test result more because it was blood-based. They also preferred the syphilis self-test because of the clarity of instructions compared with HIVST instructional material.

### Self-testing facilitators and barriers

Facilitating and deterring factors for self-testing were categorised into individual-level, community-level and structural-level factors (table 3). Convenience, privacy and autonomy were the most cited reasons why MSM preferred self-testing over facility-based testing.

**Table 3:** Summary of facilitating and deterring factors influencing MSM testing decision, including quotes from phase I in-depth interview

Social-ecological model level	Facilitators	Quotes	Barriers	Quotes
Individual	Privacy Autonomy and self-empowerment User-friendly testing and innovation High perceived trust in blood-based tests	But sometimes you need privacy because not everyone is reliable enough to keep your information with and we are humans. (on HIVST, 23-year-old MSM) One, I do the thing on my own when I’m willing to do it. Two, it produces the results that I will see on my own. (on HIVST, MSM age unspecified) It’s because it’s an improvement. Things will be better than(facility-based syphilis testing)where you go there and they take the blood. (on syphilis self-testing, 27-year-old MSM) I feel the blood based one gives a more accurate result. (on HIVST, 21-year-old MSM)	Blood sample required Reluctance to test and poor awareness surrounding syphilis	I was afraid of being pricked. (MSM, 20, on HIVST) At first, I was scared of being positive but my cousin encouraged me to go for HIV tests and told me that if I become positive that will not be the end of life. (on HIVST, 20yo MSM) I wouldn’t (consider taking a syphilis self-test) because in my mind I have already told myself that I do not have STIs, maybe I would encourage others instead. (on syphilis self-testing, 23-year-old MSM)
Community	Avoidance of social stigma High perceived importance of syphilis and peer pressure to test	The MSM community is small (…) mostly they will spread rumours that you don’t have to date this person because he has syphilis. (on syphilis self-testing, 30-year-old MSM) I have a boyfriend of mine who said he has syphilis, so he was treated for syphilis. So I need to see if I also have it. (on syphilis self-testing, 24-year-old MSM) I have a recommendation, that syphilis testing should commence immediately (…) key populations take the disease and infect their wives’ home so it becomes a vicious circle. (on syphilis self-testing, 27-year-old MSM)	Stigma at community level over testing within peer groups	You would be afraid because the people you live with, if they find out that you are self-testing for HIV, they’ll be like “what pushes you?”, which means you’re practicing something. (on HIVST, 23-year-old MSM)
Structural	Convenience and improved access Avoidance of hostility, stigma and discrimination from healthcare providers Time savings Monetary savings Avoidance of health risk for providers	No, I was just motivated with the channel of self-testing because sometimes you won’t be having any access. (on HIVST, 27-year-old MSM) You know sometimes you need to go through a whole lot of protocol to get the test kit and that’s what I wouldn’t want. (on clinic-based testing, 24-year-old MSM) I would not want to go the clinic because some nurses have got an attitude towards people like us because some of them are homophobic. (on clinic-based testing, 25-year-old MSM) I actually did it (…) without having to go anywhere or consult anyone, so yah, in terms of time, in terms of cost it was cost effective. (on HIVST, 25-year-old MSM) We do not have safety clothes to protect us when we are doing HIV tests (…) and we will be putting our lives in danger especially in this period of COVID-19. (key informant, Mabelreign Clinic)	Indefinite linkage to care and treatment availability	We give them kits, but some of them don’t come back.(…) they end up knowing your phone number they will end up not answering. So that is the challenge with the self-test. (key informant, Kuwadzana Polyclinic) On testing: ‘Going to a clinic now and again to get tested is expensive (…) you will need consultation fees which is around(3$)plus transport’. On treatment: ‘No, I had to go buy my injection and come back for administration. They only provided the syringes but for the drug I had to buy for myself’. Benzathine… rarely. Sometimes it’s out of stock and if you refer patients to go and buy outside at pharmacies, that’s where there is a challenge (key informant, Hatcliffe clinic)* (on syphilis testing, 27-year-old MSM)

*A single dose of benzathine penicillin G via intramuscular injection is the recommended treatment for syphilis in Zimbabwe. Stockouts have been reported to occur in Zimbabwe and other sub-Saharan African countries.^30^

HIVST, HIV self-testing; MSM, men who have sex with men.

### Self-testing facilitators

The following factors were facilitators for both HIVST and syphilis self-testing: privacy, autonomy and empowerment, convenience, user-friendliness, high perceived trust in blood-based tests, avoidance of social and healthcare provider stigma, monetary and time savings, and reduced contact with facility-based services in the COVID-19 context. All MSM participants felt comfortable testing alone and stated they would prefer doing their next test at home, in order to be the first to see their results. In comparison, three participants stated that facility-based testing did not provide adequate levels of privacy. MSM liked that they could conduct their test without the involvement of a healthcare provider and the convenience of it.

MSM highlighted that the lengthy waiting periods for in-facility testing are an important deterring factor. A rapid self-test could contribute to speeding up diagnosis, reducing treatment delay and interrupting more syphilis transmission. Seven participants mentioned that HIVST empowered them to test more frequently and to take control of their sexual health. All phase II participants stated that the blood draw increased their trust in the syphilis self-test. Two MSM noted the blood draw for syphilis facility-based testing is more painful than the self-test due to the nature of the self-testing lancets provided, and thus would opt for the self-test. Participants explained that they preferred the pressure-activated lancets provided in the study self-test kits, in comparison to the twist-top universal lancets used in-facility.

Participants liked that they were able to avoid being identified at a facility and stigmatised by members of their own community. Additionally, several MSM observed that self-testing prevented hostility from providers or other society members, therefore decreasing test-associated stigma. Key informants in phase I explained they valued self-testing because of the potential to reduce contact with clients, especially in the context of the COVID-19 pandemic.

### Barriers to self-testing

Themes related to barriers included the following: the challenge of self-sampling blood, reluctance to test due to poor awareness, stigma at community-level following at-home testing, indefinite linkage to care and treatment availability. Twelve participants experienced difficulty with the blood draw that they attributed to inexperience. One participant was concerned about the biohazard potential with test kit material disposal. Some MSM mentioned that self-test uptake is jeopardised among the wider community of MSM by poor awareness and the perception that they do not have STIs. MSM also expressed concerns over the fact they could be profiled or stigmatised within their own community following at-home self-testing. Participants reported that they would seek confirmatory testing if trusted information was provided on where to go and what to expect in-facility. These are legitimate concerns that align with phase I qualitative data, which showed that provider discrimination and treatment shortages exist at structural level. Key informants also reported occasional unavailability of the facility-based syphilis tests required for confirmatory testing, as these are reserved for antenatal care.

## Discussion

Our study expands on the limited literature on syphilis self-testing, includes qualitative and quantitative data, and follows MSM prior to and after self-testing. We found that syphilis self-testing was feasible and highly acceptable among MSM in Zimbabwe. The high UI (89.6%) suggests that syphilis self-testing would be acceptable in this subgroup of MSM. Overall, 12.5% of phase II MSM tested positive for syphilis, a high proportion considering the small number of participants. Participants reported self-testing was a convenient method that provided increased privacy, autonomy and diminished vulnerability in comparison to facility-based testing. The testing challenges associated with the amount of test buffer were transient and were improved by increasing the quantity of buffer provided.

Study findings are consistent with HIVST data in Zimbabwe, as well as syphilis self-testing data from China^12^ and The Netherlands.^11^ Our qualitative data suggested that many of the same facilitators and barriers for syphilis self-testing exist for HIVST. Self-testing is a private and convenient method that is preferred over facility-based testing, especially for higher-risk individuals. This is reflected in the large body of evidence that exists for HIVST, which is now well established in Zimbabwe.^23^ We found that syphilis self-testing was the first-ever syphilis test for half of our study participants. This is consistent with data from China suggesting that syphilis self-testing may increase test uptake among MSM.^12^ Recent data from HIV Self-Testing Africa (HIVSTAR) in Malawi, Zambia and Zimbabwe also show that HIVST also encourages first-time HIV testing.^24^

Our qualitative data suggest that syphilis self-testing can empower MSM to test when, where and with whom they wish. This is consistent with global HIVST qualitative literature showing how self-testing gives agency to those who test.^13 25^ Existing research also shows self-testing can improve testing frequency.^26 27^ Providing autonomy, control and creating a culture of testing among vulnerable MSM could help to build trust in the local health system, which is relatively low.^8^

One barrier to syphilis self-testing was the uncertainty of linking to confirmatory testing and treatment within health facilities. Key informants noted that Zimbabwe hospitals have variable access to non-treponemal tests and stock-outs of penicillin occur. While similar concerns existed for HIVST, linkage to care rates have been excellent.^25^ Poor linkage to syphilis care would impact the capacity for testing to translate into public health benefits for syphilis control. Embedding syphilis self-testing within the HIVST systems could be a way to enhance linkage to care. HIVST has been part of the Zimbabwe National HIV/AIDS Strategic Plan since 2016. The recent large-scale HIVSTAR implementation study found that over 75% of HIV test kit distribution in Zimbabwe was provided through community-based distribution, achieving 50.3% community-level coverage of HIVST in rural areas.^24^ A number of studies in China also show successful integration of HIV and syphilis testing services.^10^

This study has a number of limitations. First, as a mixed methods study, qualitative results should be interpreted as only an indication of the preferences of the men interviewed. The MSM participants all had at least secondary-level education, were educated about STIs and were able to access community-based services. They may therefore be early adopters within the MSM population, more likely to take up health innovations due to heightened awareness and contact with community organisations.^28^ Most participants had tried HIVST, which could have increased familiarity with the self-testing method and promoted subsequent acceptance of syphilis self-testing. The perspectives of this subset of MSM may be different from those of other, potentially more marginalised MSM in Zimbabwe. Research shows subsets of low literacy MSM have had problems using HIVST, and this may also be the case for syphilis self-testing.^29^ Another limitation is the fact that the HIV status of participants was not elicited as we wanted to allow MSM to test for syphilis without reporting this. Further research should include concurrent HIV infection as an important risk factor for syphilis infection.

This study has implications for research and policy. It has revealed that more research is needed on how we can integrate syphilis self-testing into established networks of HIVST services to facilitate implementation. Syphilis self-testing cannot effectively contribute to interrupting syphilis transmission if facility-based confirmatory testing, and treatment is not made accessible to MSM. Clinical trials are needed to assess the effectiveness and risks of syphilis self-testing in practice. From a policy perspective, many of the existing HIVST policies could be expanded to cover syphilis self-testing. Further policy development will help national leadership to embrace syphilis self-testing as a tool for expanding syphilis testing. Improving testing among key populations can reduce the bridging of syphilis into the general population, likely having an impact on the overall prevalence of syphilis, with the potential of reducing mother-to-child transmission.

In conclusion, the findings from this study suggest that syphilis self-testing may decrease user perceived test-associated stigma and empower MSM in an area where same sex relations are condemned. Innovative tools such as syphilis self-testing are needed to expand syphilis test uptake, especially for marginalised populations of MSM.

**Key messages**

- Syphilis self-testing is an empowering, innovative tool that can be used to expand uptake of STI testing among sexual minorities in Zimbabwe.
- Facilitators and barriers for syphilis self-testing are similar to those observed for HIV self-testing in Zimbabwe and other low-income and middle- income countries.
- Participants reported high self-test usability and found that self-testing provided increased privacy, convenience and autonomy in comparison to facility-based testing.

10.1136/sextrans-2020-054911.supp2 Supplementary data

10.1136/sextrans-2020-054911.supp4 Supplementary data

## Data Availability

Data are available upon reasonable request. All individual patient data collected that underlie the results reported in this article will be available (text, tables, figures and appendices). Analytical codebooks and consent forms are also available upon request. These data will be available immediately following publication to researchers who provide a methodologically sound proposal. Proposals should be directed to clarisse.sri-pathmanathan@kcl.ac.uk. To gain access, data requestors will need to sign a data access agreement.
