# Supplementary material for: Syphilis self-testing to expand test uptake among men who have sex with men: a theoretically informed mixed methods study in Zimbabwe
Source: Sex Transm Infect. 2021 Apr 29;98(3):197–202. doi: 10.1136/sextrans-2020-054911 (PMC7612625; doi:10.1136/sextrans-2020-054911)
Supplement: Supplementary data [file sextrans-2020-054911supp003.pdf]

Supplementary material 3 : Phase two post-testing in-depth interview guide for MSM

PZAT
PANGAEA ZIMBABWE AIDS TRUST

Exit interview guide for MSM field test of syphilis self-testing kits

Syphilis Self-testing to expand test uptake among men who have sex with men (SST): A pilot RCT in Zimbabwe.

About this Study:
You are invited to take part in a research study that will help us better understand whether syphilis self-testing might be a useful strategy for men having sex with men in Zimbabwe. Your participation in this project will allow us to develop better interventions to promote syphilis self-testing and linkage to care among men having sex with men across Zimbabwe.

What's Involved?
If you participate in this study, we will ask you some questions about your sociodemographic information. We will also ask you to complete a syphilis self-test, and give feedback on your experience. Your information will all be recorded using an audio recorder and the audio will not have your name on it but a study number and you will not be identified in the interview.

Is this all clear to you and are you happy for us to interview you today?

A) Participant and interviewer information

Patient ID: *E001 to E020*
(use E001 for the exit interview corresponding to the IDI with MSM001, E002 for MSM002, etc.)
Interviewer name:
Interview date:
Interview venue:

B) I would like to ask a few questions about your syphilis self-test today, and what you thought about it.

I. General usability

Q1. How was your experience with your syphilis self-test kit today?

Q2. Did you face any challenges or find any specific parts of the self-test difficult to perform?

Probe: if the participant expresses difficulty with the finger prick:
Q2.1 'Do you feel that drawing blood is a challenge you are willing to accept for this test to work?'

II. Usability index

https://journals.plos.org/plosone/article?id=10.1371/journal.pone.0227198

**STANDARD Q test usability index**	YES	NO
Did you find it easy to read/use the information sheet?		
Did you find it easy to watch/use the instructional video?		
Was it difficult for you to remove the kit components from the pack?		
Did you verify that the silica gel pouch was yellow, to confirm their test was valid for use?		
Did you remove the yellow shield from the lancet?		
Did you have difficulty lancing (pricking) their finger using the blue lancet?		
Did you have difficulty forming a blood droplet?		
Were you able to pick up a blood drop up to the black line of the capillary pipette?		
Were you able to open the green buffer bottle?		
Were you able to use the pink pipette to pick up the buffer?		
Did you drop three drops into the test device well?		
Was a control line present on the test device?		
Did you trust the self-test result?		
Did you quit the process at any point?		
Did you continue the process despite a missed or incorrect step?		

III. Instructions

Q3. Is there anything you would change in the written and pictorial instructions?

Q4. Is there anything you would change in the self-testing video?

Q5. Did you rely more on the video or the written instructions for the self-test?

IV. Attitude towards syphilis self-testing

Q6. a) Was this your first time testing for syphilis?
b) If not, please detail your experience with other forms of testing

Q7. How do you think this self-test compares to facility-based testing for syphilis? And why?

Q8. Are there any particular factors that make syphilis self-testing better than facility testing?

Q9. Are there any particular factors that make syphilis self-testing worse than facility testing?

Q10. If you had the opportunity to test for syphilis again, would you choose a self-test? Why?

Q11. Would you recommend this syphilis self-test to a friend or family member? And why?

Q12. Do you have any particular concerns regarding this syphilis self-test?

Q13. What would you change about this self-test?

V. Participant satisfaction with the syphilis self-test

Q14. a) Did you feel comfortable completing this self-test alone at home? Why?
b) If not, what were the reasons for which you felt unsafe?

Q15. Did you trust the syphilis self-test result and why?

Q16. If your result had been ***positive***, how do you think you would have reacted? [***If negative***, *ask how participant would have reacted*]

a) Would you have been able to send the result to us?
b) Would you be able to go to a health care facility to get treated? [*Ask only if positive*]
c) What kind of information and support would you have wanted in the event that you tested positive? [*Ask only if positive*]

d) . Would you disclose your test result to your sexual partner(s)?
  - If the test was negative?
  - If it was positive?

VI. Comparison to HIVST (questions reflect the MSM IDIs)

Q17. Have you used a HIV self-test before?
Q18.
a) If so, how does the syphilis self-test compare to HIV self-testing?

b) What was similar about syphilis self-testing and HIV self-testing?
c) What was different about SST and HIVST?
d) Did you find that you trusted the syphilis test result more because it is a blood-based test?

Q19. Do you have a preference between the syphilis self-test and the HIV self-test and why?

VII. SST access preferences

Q20.
a) Now that you have tried the syphilis self-test, where do you think you would prefer to collect or receive it? **Prompts**: in the post at home, through a CBO, through a health facility
b) Would you prefer to collect the self-test alone or with the help of someone?

Q21. Where would you prefer to conduct your next syphilis self- test?

Prompts:

a) Would you feel comfortable completing a syphilis self-test kit at home and why?
b) **OR** Would you prefer to do the self-test at a facility and why?

PZAT
PANGAEA ZIMBABWE
AIDS TRUST

Q22.

a) In the context of our research study, would you find it acceptable to send a photo of your completed test kit to one of our research co-ordinators for confirmation? This process would be anonymous.
b) Do you think most men in your community would have access to a smartphone to complete this confirmation process?
c) **If not**, which do you think is the best way to complete a visual confirmation of your self-test result with study coordinators?

Q23. How important do you feel it is to test for syphilis?

Q24. How do you think the availability of syphilis self-test kits would change your life?
