# Supplementary material for: Syphilis self-testing to expand test uptake among men who have sex with men: a theoretically informed mixed methods study in Zimbabwe
Source: Sex Transm Infect. 2021 Apr 29;98(3):197–202. doi: 10.1136/sextrans-2020-054911 (PMC7612625; doi:10.1136/sextrans-2020-054911)
Supplement: Supplementary data [file sextrans-2020-054911supp004.pdf]

Supplementary material 4 : Syphilis self-testing infographic distributed as part of the syphilis self - testing kit

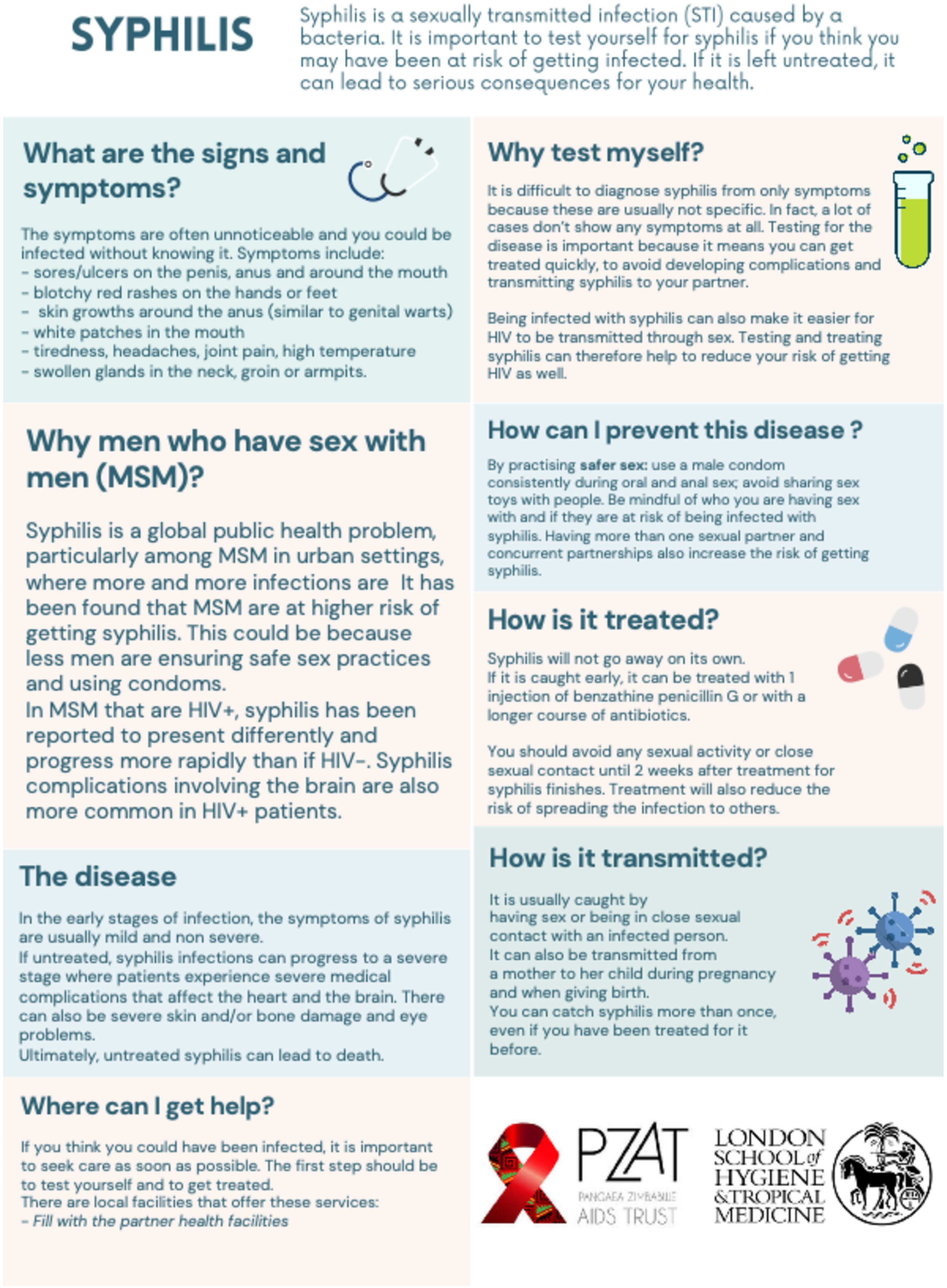

SYPHILIS SELF TESTING

When should I consider getting myself tested?

You should get tested for syphilis if:
- you have had unprotected sex with one or multiple partners.
- you are worried you might have it.
- you have symptoms of syphilis.
- a sexual partner has been diagnosed with syphilis

The test we offer is the STANDARD Q Syphilis Ab Rapid Diagnostic Test. It will test your blood for syphilis. The test can be carried out in the comfort of your own home

Why should I use a self-test?

Self-testing makes it easier for people to know their status:
- it is an easy test you can do whenever and wherever you want.
- There is no need to wait long periods of time : it is a rapid test that only takes 10 minutes.
- you do not have to attend an STI health service : it is anonymous and private.

12 easy steps

First, watch the self-test video with the QR code below and/or read the instructions from beginning to end.

1. Verify the test expiry date on the back of the packet.
2. Open the pack. Lay out the test device, capillary pipette, pink 'pipette', green 'buffer' tube, blue lancet, alcohol swab on a clean table. You can also use a phone timer.
3. Check the silica pack: if it is yellow, the test is valid for use. If the silica pack is green, you must not use this test device: please collect a new test pack.
4. Wash your hands. Then use the alcohol wipe to swipe 1 finger clean. Let air dry!
5. Use the blue lancet to prick the finger you cleaned. Use the yellow protective cap to shield the needle. Then dispose of the lancet in the empty test packet.
6. Squeeze your finger to encourage blood flow. Use the capillary pipette to pick up a drop of blood from your finger, it should exactly come up to the black line on the pipette.
7. Squeeze the drop of blood from the pipette into the square well of the test device. Dispose of the pipette in the empty test packet.
8. Add 3 drops of the green buffer into the well where you put the blood. Keep the test device flat on the table.
9. Wait for 5 minutes to read the result. Do not wait longer than 20 minutes to read the result.
10. Interpret the test results
11. Take a photo of the test result
12. Send a photo of the test result along with your patient ID number to your study coordinator.

1

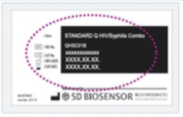

2

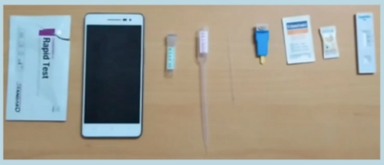

3

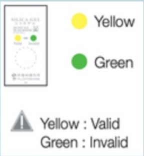

4

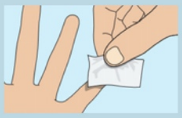

5

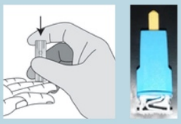

6

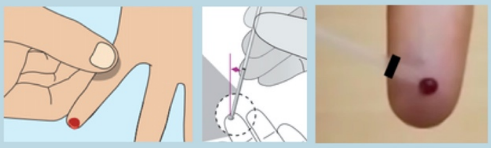

7

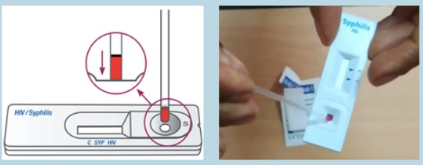

8

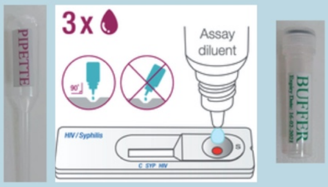

9

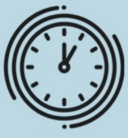

5 min

How to interpret the test

After 5 minutes the results should have appeared.
The control line 'C' should appear for all results. If the 'C' line does not appear, you will need to complete a new test.
If you obtain a 'C' line only, you are negative. If you obtain a 'C' and a 'T' line, even if it is faint, you are syphilis positive.
If you read that you are positive for syphilis, please go into a facility for a confirmation test and see a doctor as soon as possible for treatment. Please also avoid any sexual relations to avoid transmitting the disease.

10

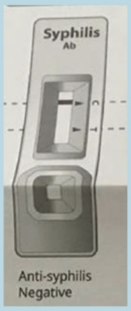

<- Negative: 1 line on 'C' ONLY

Positive -> 2 lines on 'C' AND 'T'

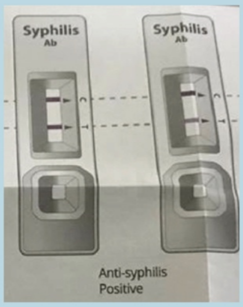

SCAN THE QR CODE TO ACCESS A VIDEO ON HOW TO USE THE SELF-TEST.

Remember that self-test kits are not 100% reliable. If you are worried about anything, get advice from a health professional. If you test positive, contact a health professional to get the medical and emotional support you need.

SST FREQUENTLY ASKED QUESTIONS

How accurate is the syphilis self-test?

The syphilis self-test is a highly accurate self-test, similar to HIV self-tests. If you follow the instructions carefully and complete the test properly, the test is 100% likely to detect syphilis, according to the test manufacturer. This is a highly sensitive testing instrument and healthcare professionals trust it to diagnose syphilis.

I am worried about the finger pricking and I do not like the sight of blood.

It is necessary for this self-test to prick your finger to obtain a few drops of your blood. Finger pricking will provoke a slightly uncomfortable pinch on your finger that will only last a second. The retractable lancet that is provided in the kit makes it easy for you to do this.

This slight pain is a downside to the test, however, it is necessary to complete the self-test properly. Finding out your syphilis status, and getting treatment if you need it, will ensure that you avoid the severe complications of syphilis.

How long does it take to complete the self-test?

The syphilis self-test is a relatively short test. The time it takes to complete the test is about 10 minutes, including 5 minutes for the test result to develop.

Is it safe for me to complete the test alone at home?

Testing for syphilis can be a stressful experience and it is a natural reaction to be anxious about it. Syphilis is an easily treatable disease so you should not be worried about your diagnosis: the treatment is straightforward and fast acting. There are also counselling services available for you following confirmatory testing and syphilis treatment.

I am worried about confidentiality as I want to keep my results private.

We assure you that maintaining confidentiality is among the most important aspects of this study. All self-test results are anonymised straightaway. Our professional research staff is highly qualified and experienced in maintaining the privacy of study participants. The study has gone through extensive ethical review to ensure that all research data remains confidential.

Should I tell my friends and family about my result?

We understand that there is stigma attached to a positive STI result. However, we want to reassure you that syphilis is not a serious disease, as it is easily treatable with a single dose of antibiotics. Early diagnosis and treatment is very beneficial to you and to your sexual partner(s). Although this may be a challenge, we also encourage you to disclose your result to your sexual partner(s). This is because if they are infected with syphilis, and you have completed your testing and treatment, they could easily re-infect you. The whole process would then have been for nothing. We highly recommend that you and your partner regularly test for STIs to avoid re-infection.

Do I need to get a second confirmatory test at a health facility?

You must only seek a second confirmatory test in the event that you read a positive result on your self-test. This confirmatory test can be done at one of our partner health facilities. However, please remember that if your self-test result is negative, you can be reassured that you are not infected with syphilis. With a negative self-test, you do not need a confirmatory test.

I am worried that the test kit will not work if I store it at home.

This self-test kit is very stable and is completely safe to use outside of health facilities. The self-test is contained in a sealed pack that can be stored in your own home and that will not lose its usability, even if it is outside of a health facility.
